# Probing an optimal class distribution for enhancing prediction and feature characterization of plant virus-encoded RNA-silencing suppressors

**DOI:** 10.1007/s13205-016-0410-1

**Published:** 2016-03-21

**Authors:** Abhigyan Nath, Karthikeyan Subbiah

**Affiliations:** Department of Computer Science, Banaras Hindu University, Varanasi, India

**Keywords:** RNA silencing, Class imbalance problem, Optimal class distribution, Balanced training set, SMOTE, Random undersampling, SVM, Relieff

## Abstract

To counter the host RNA silencing defense mechanism, many plant viruses encode RNA silencing suppressor proteins. These groups of proteins share very low sequence and structural similarities among them, which consequently hamper their annotation using sequence similarity-based search methods. Alternatively the machine learning-based methods can become a suitable choice, but the optimal performance through machine learning-based methods is being affected by various factors such as class imbalance, incomplete learning, selection of inappropriate features, etc. In this paper, we have proposed a novel approach to deal with the class imbalance problem by finding the optimal class distribution for enhancing the prediction accuracy for the RNA silencing suppressors. The optimal class distribution was obtained using different resampling techniques with varying degrees of class distribution starting from natural distribution to ideal distribution, i.e., equal distribution. The experimental results support the fact that optimal class distribution plays an important role to achieve near perfect learning. The best prediction results are obtained with Sequential Minimal Optimization (SMO) learning algorithm. We could achieve a sensitivity of 98.5 %, specificity of 92.6 % with an overall accuracy of 95.3 % on a tenfold cross validation and is further validated using leave one out cross validation test. It was also observed that the machine learning models trained on oversampled training sets using synthetic minority oversampling technique (SMOTE) have relatively performed better than on both randomly undersampled and imbalanced training data sets. Further, we have characterized the important discriminatory sequence features of RNA-silencing suppressors which distinguish these groups of proteins from other protein families.

**Electronic supplementary material:**

The online version of this article (doi:10.1007/s13205-016-0410-1) contains supplementary material, which is available to authorized users.

## Introduction

RNA silencing is a common host defense mechanism in plants against many plant RNA/DNA viruses (Li et al. 2014a; Pérez-Cañamás and Hernández 2014; Valli et al. 2001). To counter the RNA silencing defense mechanism, these plant viruses encode RNA-silencing suppressors, which disturb the host RNA silencing pathway. The molecular basis for the mechanism of encoding RNA-silencing suppressors by these plant viruses is still largely unknown. P1/HC–Pro of Potyviruses, P19 of tombusviruses and 2b proteins of cucumo-viruses are some of the well-studied RNA silencing suppressors (Qu and Morris 2005) and recently new RNA silencing suppressors are being identified in a mastrevirus (Wang et al. 2014) and in a wheat dwarf virus (Liu et al. 2014). Recent studies have also pointed to the role of suppressors in modulating the function of microRNAs (Chapman et al. 2004; Dunoyer et al. 2004).

Annotation of putative members of this family is hampered by the presence of high sequence diversity existing among these plant virus-encoded RNA-silencing suppressors (Qu and Morris 2005). The sequence similarity-based search methods like BLAST (Altschul et al. 1990) and PSI-BLAST (Altschul et al. 1997) have their inherent limitations in these situations where there exists low sequence conservation. Previously in (Jagga and Gupta 2014) the shortcomings of sequence similarity-based search methods like PSI-BLAST in correctly annotating the members of this protein family are emphasized. Machine learning methods trained on mathematically represented suitable input feature vectors become a viable alternative to sequence similarity-based search methods. Previously different machine learning methods have been successfully applied to solve biological classification tasks (Kumari et al. 2015; Nath et al. 2012; Nath and Subbiah 2014). But the true performance of machine learning methods is affected by various factors such as class imbalance (Nath and Subbiah 2015a), imperfect learning due to some missing example instances and selection of inappropriate input features.

The class imbalance problem is quite common in biological datasets, where there is a huge difference in the number of instances belonging to the different classes and subclasses. These types of imbalanced datasets result in classifier bias towards the majority class and tend to produce majority class classifier (Wei and Dunbrack 2013). In most of the cases, the class of interest is the minority class and is the cause for lower sensitivity. Many methods had been proposed to deal with the class imbalance problem. Previously it has been stressed that the natural class distribution may not be optimal for training (Lee 2014; Weiss and Provost 2003) and the requirement of a balanced training set for proper learning has been pointed out by Dunbrack et al. (Wei and Dunbrack 2013). In the current work, we propose a technique to achieve better learning of both the positive and negative classes by experimenting with different resampling methods to balance the dataset with varying degree of class distributions. We have also repeated the experiments on different machine learning algorithms on imbalanced, Synthetic Minority Oversampling Technique (SMOTE) (Chawla et al. 2002) oversampled and randomly undersampled datasets to find the optimal class distribution. We used the sequence features like amino acid composition, property group composition, dipeptide counts and property group n-grams for creating the input feature vectors. Broadly, two types of approaches are used for handling the class imbalance, (1) resampling methods which are algorithm independent and are transferable to different machine learning algorithms and (2) internal approaches which involve altering the existing algorithms and its various parameters for adapting to imbalance class distribution. The SMOTE and random undersampling fall under resampling methods, although other sophisticated varieties of SMOTE exist (Barua et al. 2014; Han et al. 2005; Nakamura et al. 2013), but in the present study, we have limited our focus on simple undersampling and SMOTE oversampling as they are found to be useful for many classifiers (Blagus and Lusa 2013) and in many biological classification problems (Batuwita and Palade 2009; MacIsaac et al. 2006; Xiao et al. 2011).

The current method explored the possibility of improvement in prediction accuracy of the machine learning algorithms using optimal class distribution and presented in detail the behavior of the tested learning algorithms with varying degrees of resampling. From the current work, it is also proved that prediction accuracy for the plant virus-encoded RNA-silencing suppressor proteins can be improved using resampling techniques.

## Materials and methods

### Dataset

We have used the dataset as used in (Jagga and Gupta 2014) which consisted of 208 plant virus-encoded RNA-silencing suppressor proteins (RSSPs) belonging to positive class and 1321 non-suppressor proteins (NSPs) belonging to negative class, for this study. The CD-HIT (Li and Godzik 2006) was applied separately to these classes of sequences to reduce the redundancy at 70 % sequence identity. Here, the positive class is the minority class as the number of positive class sequences is relatively very small when compared to the number of negative class sequences and their prediction will suffer from the imbalance class factor.

### Extraction of feature vectors

The quality of the attributes of the protein sequences selected for creating the input feature vector will have great influence in learning the concepts of a particular protein family. We represented each protein sequence as the combination of following sequence features to create input instances and they are explained below.

#### Amino acid composition feature

Different proteins are evolved through the avoidance and preference of some specific amino acids and leads to some certain unique set of percentage frequency composition, which can be used successfully for discriminatory purposes (Nath and Subbiah 2014). So we have taken the frequency percentage of distribution of the 20 amino acids along the length of the protein sequence as one of the features for creating the input feature vector. It is calculated using the following formula:

1 $$ {\text{AA}}_{i} = \frac{{{\text{TC}}_{{{\text{AA}},i}} }}{{{\text{TC}}_{{{\text{res}}, i}} }} \times 100, $$

where AA denotes for one of the 20 amino acid residues, AA_*i*_ denotes the amino acid percentage frequency of specific type ‘AA’ in the *i*th Sequence, TC_AA,*i*_ denotes the total count of amino acid of specific ‘AA’ type in the *i*th sequence, TC_res,*i*_ denotes the total count of all residues in the *i*th sequence (i.e., sequence length).

#### Amino acid property group composition feature

The amino acids can be grouped according to their physicochemical properties. The Table 1 contains the list of amino acids belonging to the 11 different physicochemical groups. We have taken the percentage frequency composition of the 11 different amino acid property groups as used in (Nath et al. 2013) as the second feature. The formula for calculating this feature attribute is given below.

**Table 1:** Physicochemical groupings of amino acids taken for the present study

S. no.	Name of amino acid property group	Amino acids in the specific group
1.	Tiny amino acids group	Ala, Cys, Gly, Ser, Thr
2.	Small amino acids group	Ala, Cys, Asp, Gly, Asn, Pro, Ser, Thr and Val
3.	Aliphatic amino acids group	Ile, Leu and Val
4.	Nonpolar amino acid groups	Ala, Cys, Phe, Gly, Ile, Leu, Met, Pro, Val, Trp and Tyr
5.	Aromatic amino acid group	Phe, His, Trp and Tyr
6.	Polar amino acid group	Asp, Glu, His, Lys, Asn, Gln. Arg, Ser, and Thr
7.	Charged amino acid group	Asp, Glu, His, Arg, Lys
8.	Basic amino acid group	His, Lys and Arg
9.	Acidic amino acid group	Asp and Glu
10.	Hydrophobic acid group	Ala, Cys, Phe, Ile, Leu, Met, Val, Trp, Tyr
11.	Hydrophilic acid group	Asp, Glu, Lys, Asn, Gln

2 $$ {\text{PG}}_{i} = \frac{{{\text{TC}}_{{{\text{PG}}, i}} }}{{{\text{TC}}_{{{\text{res}}, i}} }} \times  1 0 0 , $$

where PG denotes one of the 11 different amino acid property groups, PG_*i*_ denotes the percentage frequency of specific ‘PG’ amino acid property group in the *i*th sequence, TC_PG,*i*_ denotes the total count of specific amino acid property group ‘PG’ in the *i*th sequence, TC_res,*i*_ denotes the total count of all residues in the *i*th sequence.

#### Dipeptide counts

There are four hundred different possible dipeptides from 20 amino acids. To take advantage of the local sequence order and amino acid coupling into the prediction we have taken the dipeptide counts as the third feature.

#### Property group *n*-grams

To take into the conservation of similar contiguous physicochemical amino acid property groups in the protein sequence, we have calculated the property groups *n*-grams, where n is the window length. In the current work we have taken the window length of 2 as the fourth feature and is calculated by the formula given below:

3 $$ {\text{Physicochemical}}\,\; 2 {\text{-grams}}:\,\;{\text{Small}} = \sum\limits_{i = 1}^{N - 1} {C\left({i,i + 1} \right)}, $$

where *N* denotes the length of the protein sequence, *i* denotes the position of the amino acid residue along the protein sequence, if the condition $({\text{aa}}_{i} \in S^{*}   {\text{and aa}}_{i + 1} \in S^{*} )$ is true then $C(i,i + 1)$ = 1 else $C(i,i + 1)$ = 0 where the set of small aminoacids S^*^ = {Ala,Cys,Asp,Gly,Asn,Pro,Ser,Thr,Val}.

The above formula is used to calculate physicochemical 2-grams for the small amino acid group. In the similar way the physicochemical 2-grams for the other ten physicochemical property groups were calculated. An example feature vector is provided in Supplementary Table S1–S3.

### Optimal balancing protocol

#### SMOTE

It was proposed by Chawla et al. (2002) for intelligent oversampling of minority samples as opposed to random oversampling, which may bias the learning towards the overrepresented samples. It is a nearest neighbor-based method, where it first chooses k nearest samples for a particular minority sample. It then randomly selects the j minority samples to create a synthetic minority sample. Successful use of SMOTE in classification tasks have been shown in (Li et al. 2014b; Nath and Subbiah 2015b; Suvarna Vani and Durga Bhavani 2013).

#### Classification protocol SVM

Support vector machines are supervised learning algorithms and are based on statistical learning theory of Vapnik (Vapnik 1995, 1998). Previous usage of SVM for biological classification/prediction problems has found them to be more accurate and also they are robust to noise and well suited for high dimensional datasets (Kandaswamy et al. 2011; Mishra et al. 2014; Pugalenthi et al. 2010). We have used the sequential minimization optimization (SMO) (Platt 1999) algorithm for fast training of SVM with polynomial kernel with an exponent value of 1 and *C* = 1 (a complexity parameter which SMO uses to build the hyperplane between the two classes, -C governs softness of the class margins).

**Fig. 1:**
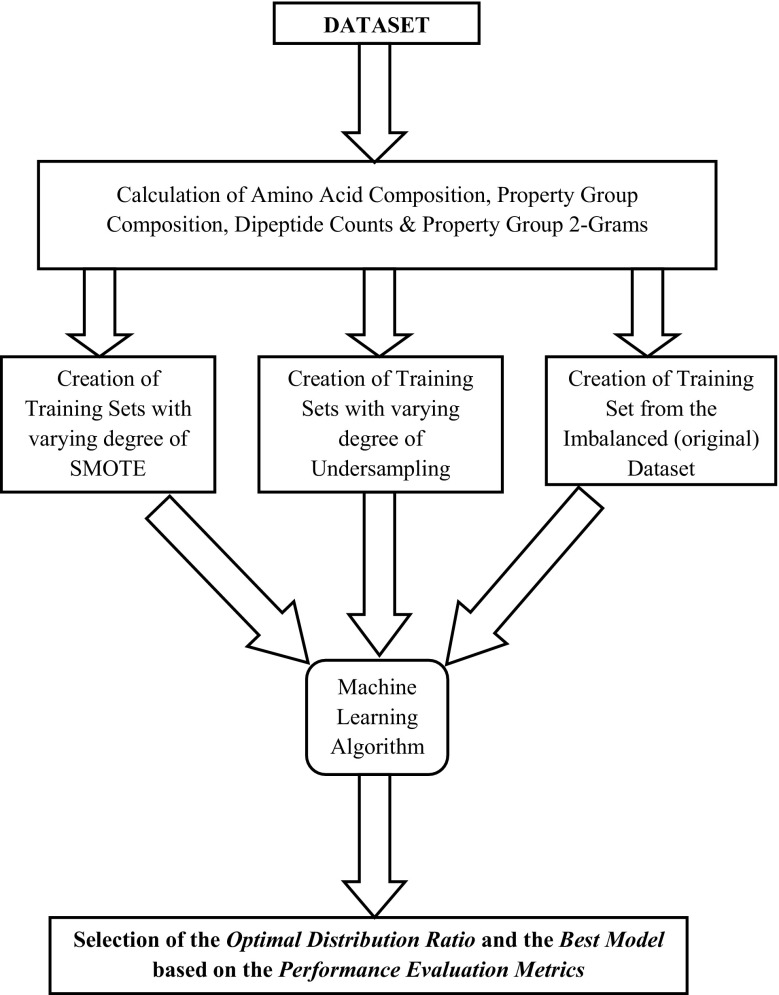
Schematic representation of the current pipeline

All the experiments were carried out using WEKA (Hall et al. 2009) which is an open source java-based machine learning platform. The schematic representation of the current methodology is given in Fig. 1.

### Characterization of plant virus-encoded RNA-silencing suppressors

We have used Relieff (Kira and Rendell 1992) feature ranking algorithm to rank the sequence features according to their discriminating ability. Relieff is a nearest neighbor-based feature relevance algorithm. It starts by randomly selecting an instance and then searches for the nearest neighboring instances belonging to the same and opposite classes. It compares the attributes of the instance with its nearest neighbors and assigns weights according to its discriminating ability.

### Performance evaluation metrics

We have used stratified tenfold cross validation for the evaluation of the various models. The performances of the machine learning algorithms were assessed with both threshold-dependent and threshold-independent parameters. These parameters are derived from the values of the confusion matrix, namely TP: true positive that is the number of correctly predicted RSSPs, TN: true negative that is the number of correctly predicted NSPs, FP: false positive that is the number of incorrectly predicted NSPs and FN: false negative that is the number of incorrectly predicted RSSPs. The formula for calculating the evaluation parameters are given below:

*Sensitivity* Expresses the percentage of correctly predicted RSSPs.

4 $$ {\text{Sensitivity}} = {{\text{TP}} \mathord{\left/ {\vphantom {{\text{TP}} {\left( {{\text{TP}} + {\text{FN}}} \right)}}} \right. \kern-0pt} {\left( {{\text{TP}} + {\text{FN}}} \right)}} \times 100. $$

*Specificity* Expresses the percentage of correctly predicted NSPs.

5 $$ {\text{Specificity}} = {{\text{TN}} \mathord{\left/ {\vphantom {{\text{TN}} {\left( {{\text{TN}} + {\text{FP}}} \right)}}} \right. \kern-0pt} {\left( {{\text{TN}} + {\text{FP}}} \right)}} \times 100. $$

*Accuracy* Expresses the percentage of both correctly predicted RSSPs and NSPs.

6 $$ {\text{Accuracy}} = {{\left( {{\text{TP}} + {\text{TN}}} \right)} \mathord{\left/ {\vphantom {{\left( {{\text{TP}} + {\text{TN}}} \right)} {\left( {{\text{TP}} + {\text{FP}} + {\text{TN}} + {\text{FN}}} \right)}}} \right. \kern-0pt} {\left( {{\text{TP}} + {\text{FP}} + {\text{TN}} + {\text{FN}}} \right)}} \times 100. $$

*AUC* Area under the receiver operating characteristic (ROC) curve that summarizes the ROC by a single numerical value. It is a threshold-independent metric and can take values from 0 to 1 (Bradley 1997). The value of 0 indicates the worst case, 0.5 for random ranking and 1 indicates the best prediction.

*Youden’s Index* This performance metric evaluates the algorithm’s ability to avoid failure. Lower failure rates are expressed by higher index values (Youden 1950). It is calculated as:

7 $$ Y = \left( {\text{Sensitivity}} \right) - \left( {1 - {\text{Specificity}}} \right). $$

*Dominance* It expresses the relationship between the TP_rate (true-positive rate) and TN_rate (true-negative rate) and is proposed by (García et al. 2009). It is calculated as:

8 $$ {\text{Dominance}} = \left({\text{TP}} \_{\text{rate}}\right)-\left({\text{TN}} \_{\text{rate}}\right). $$

Its value ranges from −1 to +1. A dominance value of +1 means a perfect accuracy on the positive class and a value −1 means a perfect accuracy on the negative class. A value closer to zero means a balance between TP_rate and TN_rate.

*g*-*mean*: it was proposed by Kubat et al. (1997), this evaluation parameter shows the balance between sensitivity and specificity. It is the geometric mean of sensitivity and specificity. It is calculated as:

9 $$ g{\text{-means}} = \sqrt {{\text{Sensitivity}} \times {\text{Specificity}}}. $$

## Results and discussion

We have experimented with four different machine learning algorithms, namely—(1) naive Bayes (NB), (2) Fischer linear discriminant function (implemented as FLDA in WEKA), (3) support vector machines with sequential minimization optimization (SMO) and (4) K nearest neighbor (implemented as IBK in WEKA) on the imbalanced dataset (original), randomly undersampled dataset (with varying class distribution) and SMOTE oversampled dataset (with varying class distribution) to find the optimal class distribution for each of these classifiers.

### Learning performance on imbalanced dataset

Observing the values of the performance evaluation parameters obtained from the different machine learning algorithms when trained with the imbalanced dataset (Table 2), the overall accuracy of SMO and IBK crossed above 90 %, although with a large difference in their individual accuracies for the positive (sensitivity) and negative classes (specificity), respectively. The training on the imbalanced dataset resulted in high specificity values for all the learning algorithms except the naive Bayes. The negative dominance values of all the learning algorithms (except the naive Bayes) are also biased towards the TN_rate. This indicates that optimal learning with higher accuracies (sensitivity and specificities) for the positive and negative classes is difficult in cases where there is an imbalance between the positive and negative class instances.

**Table 2:** Performance evaluation metrics of the different learning algorithms trained on the imbalanced datasets

Learning algorithms	Sensitivity	Specificity	Accuracy	AUC	Youden’s Index	Dominance	*g*-means
Imbalanced data set							
NB	90.8	29.2	36.9	0.678	0.200	0.616	51.49
FLDA	64.7	84.9	82.3	0.819	0.492	−0.202	74.1
SMO	52.1	97.1	91.4	0.746	0.496	−0.450	71.1
IBK	68.9	97.0	93.4	0.841	0.659	−0.281	81.7

### Learning performance on randomly undersampled datasets

Nearest neighbor-based IBK method performed better than all the other machine learning algorithms and closely followed by SMO, when the original imbalance dataset was subjected to undersampling at different distribution rates for dealing with the data imbalance problem. The values of different performance evaluation parameters obtained by different degrees of class distribution are recorded in the Table 3. When the dataset is fully balanced by undersampling (undersampled 1:1), we obtained higher accuracy for the positive class samples than all other undersampled datasets. Highest overall accuracy of 91.8 % is obtained by IBK when the undersampling rate is 1:5 closely followed by SMO with 89.4 % accuracy. In the case of the undersampling datasets, IBK performed better than all other machine learning algorithms.

**Table 3:** Performance evaluation metrics of the different machine learning algorithms trained on the different randomly undersampled training sets

Learning algorithms	Sensitivity	Specificity	Accuracy	AUC	Youden’s Index	Dominance	*g*-means
Undersampling (1:1) (fully balanced) training set							
NB	91.6	23.5	57.6	0.631	0.151	0.681	46.3
FLDA	73.9	68.5	71.4	0.768	0.424	0.054	71.1
SMO	77.3	74.8	76.1	0.761	0.521	0.025	76.0
IBK	80.7	81.5	81.1	0.818	0.622	−0.008	81.5
Undersampling (1:2) training set							
NB	89.1	30.3	49.9	0.666	0.194	0.588	51.9
FLDA	63.0	63.0	63.0	0.661	0.26	0	63
SMO	72.3	88.7	83.2	0.805	0.61	−0.164	80.08
IBK	72.3	90.8	84.6	0.809	0.631	−0.185	81.0
Undersampling (1:3) training set							
NB	90.8	28.9	44.3	0.664	0.197	0.619	51.2
FLDA	58.8	55.7	56.5	0.613	0.507	0.031	57.2
SMO	67.2	91.9	85.7	0.796	0.591	−0.247	78.5
IBK	72.3	93.0	87.8	0.082	0.653	−0.207	81.9
Undersampling (1:4) training set							
NB	88.2	31.1	42.5	0.694	0.193	0.571	52.37
FLDA	64.7	73.5	71.8	0.731	0.382	−0.088	68.9
SMO	63.0	92.4	86.6	0.777	0.554	−0.294	76.2
IBK	68.9	94.7	89.6	0.823	0.636	−0.258	80.7
Undersampling (1:5) training set							
NB	89.1	31.1	40.8	0.692	0.202	0.58	52.6
FLDA	66.4	79.0	76.9	0.791	0.454	−0.126	72.42
SMO	57.1	94.6	88.4	0.759	0.61	−0.375	73.4
IBK	70.6	93.9	90.1	0.841	0.645	−0.233	81.4
Undersampling (1:6) training set							
NB	89.1	29.6	38.1	0.688	0.187	0.595	51.3
FLDA	68.1	80.4	78.6	0.805	0.485	−0.123	73.9
SMO	56.3	95.0	89.4	0.756	0.513	−0.387	73.13
IBK	71.4	95.2	91.8	0.824	0.666	−0.238	82.4

### Learning performance on SMOTE oversampled datasets

**Fig. 2:**
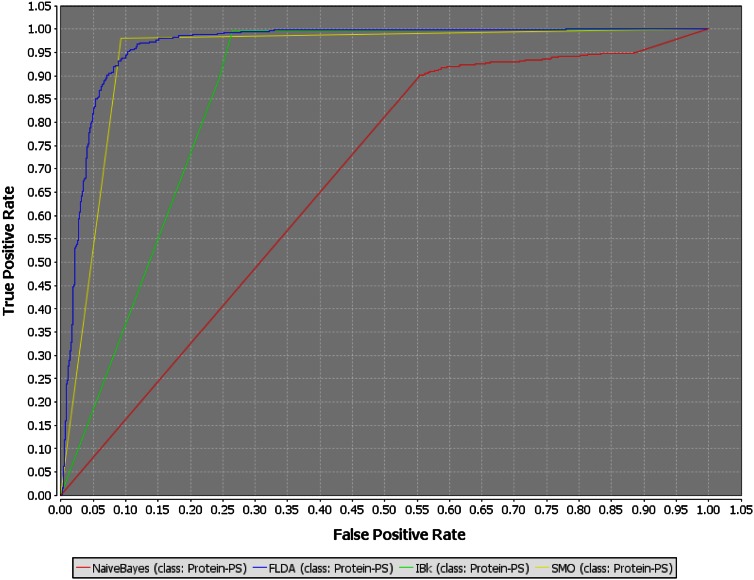
ROC curves of the four classifiers using the training set with optimal class distribution [SMOTE (500 %)]

SMO performed better than all the other machine learning algorithms closely followed by FLDA on SMOTE oversampled datasets. The values of different performance parameters are recorded in the Table 4. One of the best noticeable effects of oversampling is the immediate increase in sensitivity values for all the four machine learning algorithms. There is a regular increasing trend for the Youden’s Index (which shows the model’s ability to avoid faults) with increasing rate of SMOTE oversampling. The best trade-off for the different evaluation parameters was obtained for the SMOTE 500 % dataset with SMO as the machine learning algorithm. This particular training dataset gave the best performance evaluation metrics with SMO as the learning algorithm. With this training dataset we could achieve 98.5 % sensitivity, 92.6 % specificity, 95.3 % overall accuracy, and 0.955 of AUC. A high value of sensitivity indicates that the model is very accurate for the positive minority class samples. A positive dominance index of 0.059 also confirms the fact that the model is good in predicting minority samples. A high value of the Youden’s Index (0.911) indicates the model’s superiority in fault avoidance ability. A *g*-means value of 95.5 also indicates an optimal balance between sensitivity and specificity. ROC plots for the four different machine learning algorithms trained on the best performing training set (SMOTE oversampled 500 % dataset) are shown in Fig. 2.

**Table 4:** Performance evaluation metrics of the different machine learning algorithms trained on the different SMOTE oversampled training sets

Learning Algorithms	Sensitivity	Specificity	Accuracy	AUC	Youden’s Index	Dominance	*g*-means
SMOTE 100 % training set							
NB	91.2	33.1	46.1	0.738	0.243	0.581	54.9
FLDA	81.5	84.5	83.8	0.896	0.660	−0.030	82.9
SMO	81.1	94.7	91.6	0.879	0.758	−0.136	87.6
IBK	97.9	85.1	88.0	0.912	0.830	0.128	91.2
SMOTE 200 % training set							
NB	91.6	35.0	52.1	0.749	0.266	0.566	56.6
FLDA	91.3	85.4	87.2	0.934	0.767	0.005	88.3
SMO	92.4	93.9	93.5	0.932	0.863	−0.015	93.1
IBK	98.9	79.7	85.5	0.894	0.786	0.192	88.7
SMOTE 300 % training set							
NB	91.2	36.0	56.1	0.751	0.272	0.552	57.2
FLDA	95.2	84.4	88.3	0.946	0.796	0.108	89.6
SMO	96.2	92.3	93.7	0.942	0.885	0.003	94.2
IBK	99.4	79.1	86.5	0.890	0.785	0.203	88.6
SMOTE 400 % training set							
NB	90.9	36.9	56.1	0.751	0.278	0.54	57.9
FLDA	95.8	84.9	89.4	0.952	0.807	0.109	90.1
SMO	96.5	91.8	93.7	0.941	0.883	0.047	94.1
IBK	99.3	74.6	84.9	0.870	0.733	0.247	86.0
SMOTE 500 % training set							
NB	92.0	36.8	62.4	0.745	0.288	0.552	58.1
FLDA	97.3	83.7	90.0	0.962	0.810	0.136	90.2
SMO	98.5	92.6	95.3	0.955	0.911	0.059	95.5
IBK	99.6	73.8	85.8	0.867	0.734	0.258	85.7
SMOTE 594 % (fully balanced) training set							
NB	92.4	36.4	64.4	0.742	0.288	0.56	57.9
FLDA	97.7	85.1	91.4	0.964	0.828	0.12	91.1
SMO	97.9	90.8	94.4	0.944	0.887	0.071	94.2
IBK	99.6	73.5	86.6	0.862	0.731	0.261	85.5

To further validate the learned models trained on a SMOTE oversampled dataset (500 %), we have used leave on out cross validation test (Chou and Zhang 1995). It is deemed as the most objective and robust test and has been used by many researchers for the assessment of classifier models (Chou and Cai 2004; Gao et al. 2005; Xie et al. 2013), the results are given in Table 5.

**Table 5:** Leave on out cross validation performance evaluation metrics on the best training set

Learning algorithms	Sensitivity	Specificity	Accuracy	AUC	Youden’s Index	Dominance	*g*-means
LOOCV on SMOTE (500 %)							
NB	92.3	36.4	62.3	0.745	0.287	0.559	57.96
FLDA	97.2	85.1	90.7	0.966	0.823	0.121	90.90
SMO	98.9	92.3	95.3	0.956	0.912	0.066	95.50
IBK	99.4	75.8	86.8	0.876	0.752	0.236	86.80

Further, a corrected resampled paired *t* test was performed using WEKA with SMO as the baseline classifier. The *t* test was performed at the 5 % significance level. Each tenfold cross validation was repeated ten times (10 × 10 runs for each algorithm). Percentage correctly predicted instances, AUC, TP rate and TN rate was used for comparison with *t* test. The results of the *t* test are provided in the supplementary material (Table S4a–d).

### Comparing the results with previous study

We have compared the evaluation metric of the current study with the previous study and the performance evaluation metric values for the current best training set and the previously reported values are presented in Table 6.

**Table 6:** Comparison of the performance evaluation metrics of the current work with the previous methods

Methods	Sensitivity	Specificity	Accuracy	AUC	Youden’s Index	Dominance	*g*-means
Jagga and Gupta (2014)	80.90	80.57	80.61	0.910	0.614	0.003	80.70
SMO [SMOTE (500 %)]	98.50	92.60	95.30	0.955	0.911	0.059	95.50

On comparison with the previous method, the current SMOTE (500 %) model achieved far better performance evaluation metrics.

It is also observed that both the SMOTE oversampling and random undersampling have least effect on the performance of the naive Bayes algorithm, a similar observation has also been made by (Daskalaki et al. 2006).

### Characterization of RNA-silencing suppressors using sequence-based features

**Fig. 3:**
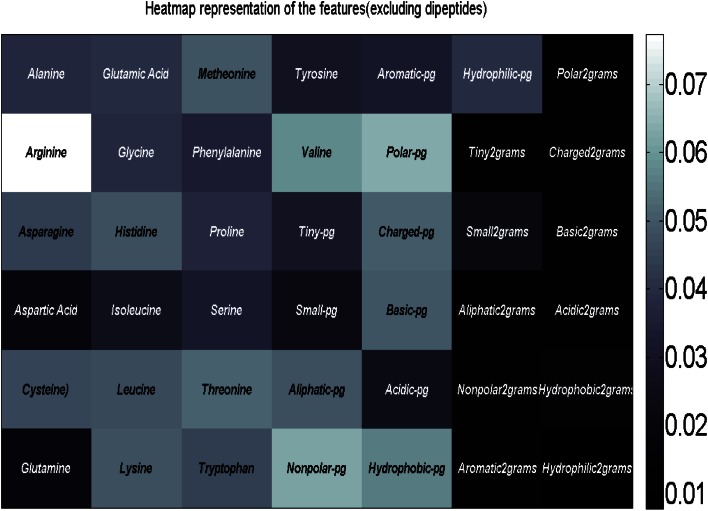
Heat map representation of ranking the sequence features (excluding dipeptides) according to their discriminative ability

**Fig. 4:**
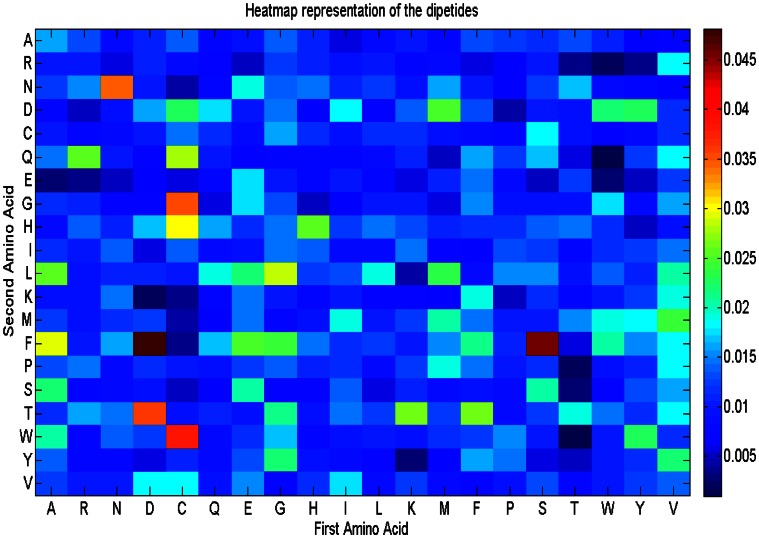
Heat map representation of ranking the dipeptides according to their discriminative ability

In Fig. 3, we have plotted the heat map representation of the sequence attributes except the dipeptides. Figure 4 presents the heat map representation of the dipeptides. The color bar in both the figures (on the right side of both the figures) shows the color intensity proportional to the feature ranking scores which are calculated according to their discriminating ability. Observing the Fig. 3, arginine, polar and nonpolar property groups are the most useful discriminatory features. From Fig. 4, it can also be observed that DF, SF, NN, DT, CW, CG are the most discriminatory dipeptides.

Arginines are relatively important in binding sites (Barnes 2007), also it is imperative to mention the importance of the role of arginine in suppressor activity of PRS suppressor (2b) of a cucumber mosaic virus strain (CM95R) (Goto et al. 2007) where it facilitates in binding to RNA and in potato virus M where mutational studies have shown the importance of arginines in suppression activity (Senshu et al. 2011). The importance of nonpolar amino acids, specifically isoleucine in suppression activity is also emphasized in (Carr and Pathology 2007).

## Conclusions

Machine learning-based approaches are apposite techniques when compared to sequence alignment-based methods for the prediction of plant virus-encoded RNA-silencing suppressors and can become the superior alternative if the imbalance dataset problem is properly resolved. The protein family classification problem intrinsically presents a class imbalance situation, where the class of interest is a particular protein family which constitutes the positive class and the rest of the protein families belonging to the negative classes. Naturally, there is a large difference between the number of instances belonging to positive and negative classes. Depending on the mathematical representation of the protein sequences, machine learning-based approaches can capture the hidden relationship among the calculated protein attributes, which is most of the times better than alignment-based methods for protein classification. The plant virus-encoded RNA-silencing suppressor protein classification presents a data imbalance problem; we compared the learning of different machine learning algorithms on imbalanced, SMOTE oversampled and randomly undersampled datasets. The results reported in this study showed that learning is non-optimal for imbalanced positive and negative class data sets. The behavior of the machine learning algorithms is different in SMOTE oversampling and random undersampling. IBK performed better on randomly undersampled datasets, while the performance of SMO is superior to all other machine learning algorithms on SMOTE oversampled datasets. Better performance evaluation metrics were obtained on SMOTE oversampled datasets than on the randomly undersampled datasets. The best model is achieved with SMOTE oversampling when SMO is used as the learning algorithm. This also points to the fact that the full (ideal) balancing between the positive and negative classes may not fully eliminate the classifier bias. The current study supports and provides evidence to the fact that the learning of different machine learning algorithms can be improved using an optimal class distribution and also the fully balanced class distribution need not be optimal for the training of the learning algorithms. Individual accuracies and learning on the positive and negative classes can be increased by changing the class distribution. Overall the performance of the various machine learning algorithms on SMOTE oversampled datasets is better than the random undersampled datasets. Further, we have ranked the calculated sequence features according to their discriminating ability in classifying plant virus-encoded RNA-silencing suppressors from non-suppressors. The current pipeline can be successfully applied to other protein family classification problem with different degrees of imbalance. The current method explored the possibility of improvement in prediction accuracy of the four machine learning algorithms using an optimal class distribution that provides the best trade-off between imbalance dataset and the diversity of the dataset. A comprehensive study was carried out and presented in detail the behavior of the tested learning algorithms with varying degrees of resampling. It is also proved that prediction accuracy for the plant virus suppressor proteins can be improved using the optimal class distribution ratio.

Future research can be carried out by incorporating additional diversifying techniques to deal with the related problem of incomplete learning. More sophisticated techniques can be evolved to deal with the trade-off between the balancing factor and input instance diversity. Further research in this direction can lead to the formulation of some kind of standard in creating benchmark data sets to every specific biological problem.

## Electronic supplementary material

Below is the link to the electronic supplementary material.

Supplementary material 1 (DOCX 19 kb)

## References

[CR1] Altschul SF, Gish W, Miller W, Myers EW, Lipman DJ (1990). Basic local alignment search tool. J Mol Biol.

[CR2] Altschul S, Madden T, Schaffer A, Zhang J, Zhang Z, Miller W, Lipman D (1997). Gapped BLAST and PSI-BLAST: a new generation of protein database search programs. Nucleic Acids Res.

[CR3] Barnes MR (2007) Bioinformatics for geneticists: a bioinformatics primer for the analysis of genetic data. Wiley

[CR4] Barua S, Islam MM, Xin Y, Murase K (2014). MWMOTE—majority weighted minority oversampling technique for imbalanced data set learning knowledge and data engineering. IEEE Trans.

[CR5] Batuwita R, Palade V (2009). microPred: effective classification of pre-miRNAs for human miRNA gene prediction. Bioinformatics.

[CR6] Blagus R, Lusa L (2013). SMOTE for high-dimensional class-imbalanced data. BMC Bioinform.

[CR7] Bradley AP (1997). The use of the area under the ROC curve in the evaluation of machine learning algorithms. Pattern Recogn.

[CR8] Carr T, Pathology ISUP (2007) Genetic and molecular investigation of compatible plant-virus interactions. Iowa State University, Iowa

[CR9] Chapman EJ, Prokhnevsky AI, Gopinath K, Dolja VV, Carrington JC (2004). Viral RNA silencing suppressors inhibit the microRNA pathway at an intermediate step. Genes Dev.

[CR10] Chawla NV, Bowyer KW, Hall LO, Kegelmeyer WP (2002). SMOTE: synthetic minority over-sampling technique. J Artif Int Res.

[CR11] Chou K-C, Cai Y-D (2004). Predicting protein structural class by functional domain composition. Biochem Biophys Res Commun.

[CR12] Chou K, Zhang C (1995). Prediction of protein structural classes. Crit Rev Biochem Mol Biol.

[CR13] Daskalaki S, Kopanas I, Avouris NM (2006). Evaluation of classifiers for an uneven class distribution problem. Appl Artif Intell.

[CR14] Dunoyer P, Lecellier CH, Parizotto EA, Himber C, Voinnet O (2004). Probing the microRNA and small interfering RNA pathways with virus-encoded suppressors of RNA silencing. Plant Cell.

[CR15] Gao Y, Shao S, Xiao X, Ding Y, Huang Y, Huang Z, Chou KC (2005). Using pseudo amino acid composition to predict protein subcellular location: approached with Lyapunov Index, Bessel function, and Chebyshev filter. Amino Acids.

[CR16] García V, Mollineda RA, Sánchez JS (2009) Index of balanced accuracy: a performance measure for skewed class distributions. In: Araujo H, Mendonça A, Pinho A, Torres M (eds) Pattern recognition and image analysis, vol 5524. Lecture notes in computer science. Springer, Heidelberg, pp 441–448. doi:10.1007/978-3-642-02172-5_57

[CR17] Goto K, Kobori T, Kosaka Y, Natsuaki T, Masuta C (2007). Characterization of silencing suppressor 2b of cucumber mosaic virus based on examination of its small RNA-binding abilities. Plant Cell Physiol.

[CR18] Hall M, Frank E, Holmes G, Pfahringer B, Reutemann P, Witten IH (2009). The WEKA data mining software: an update. SIGKDD Explor Newsl.

[CR19] Han H, Wang W-Y, Mao B-H (2005) Borderline-SMOTE: a new over-sampling method in imbalanced data sets learning. In: Huang D-S, Zhang X-P, Huang G-B (eds) Advances in intelligent computing, vol 3644. Lecture notes in computer science. Springer, Heidelberg, pp 878–887. doi:10.1007/11538059_91

[CR20] Jagga Z, Gupta D (2014). Supervised learning classification models for prediction of plant virus encoded RNA silencing suppressors. PLoS ONE.

[CR21] Kandaswamy K, Pugalenthi G, Hazrati M, Kalies K-U, Martinetz T (2011). BLProt: prediction of bioluminescent proteins based on support vector machine and relief feature selection. BMC Bioinformatics.

[CR22] Kira K, Rendell LA (1992) A practical approach to feature selection. Paper presented at the proceedings of the ninth international workshop on machine learning, Aberdeen

[CR23] Kubat M, Holte R, Matwin S (1997) Learning when negative examples abound. In: van Someren M, Widmer G (eds) Machine learning: ECML-97, vol 1224. Lecture notes in computer science. Springer, Heidelberg, pp 146–153. doi:10.1007/3-540-62858-4_79

[CR24] Kumari P, Nath A, Chaube R (2015). Identification of human drug targets using machine-learning algorithms. Comp Biomed.

[CR25] Lee PH (2014). Resampling methods improve the predictive power of modeling in class-imbalanced datasets. Int J Environ Res Public Health.

[CR26] Li W, Godzik A (2006). Cd-hit: a fast program for clustering and comparing large sets of protein or nucleotide sequences. Bioinformatics.

[CR27] Li F, Huang C, Li Z, Zhou X (2014). Suppression of RNA silencing by a plant DNA virus satellite requires a host calmodulin-like protein to repress *RDR6* expression. PLoS Pathog.

[CR28] Li H, Pi D, Wang C (2014). The prediction of protein-protein interaction sites based on RBF classifier improved by SMOTE. Math Probl Eng.

[CR29] Liu Y, Jin W, Wang L, Wang X (2014). Replication-associated proteins encoded by wheat dwarf virus act as RNA silencing suppressors. Virus Res.

[CR30] MacIsaac KD (2006). A hypothesis-based approach for identifying the binding specificity of regulatory proteins from chromatin immunoprecipitation data. Bioinformatics.

[CR31] Mishra NK, Chang J, Zhao PX (2014). Prediction of membrane transport proteins and their substrate specificities using primary sequence information. PLoS ONE.

[CR32] Nakamura M, Kajiwara Y, Otsuka A, Kimura H (2013). LVQ-SMOTE—learning vector quantization based synthetic minority over-sampling technique for biomedical data. BioData Min.

[CR33] Nath A, Subbiah K (2014). Inferring biological basis about psychrophilicity by interpreting the rules generated from the correctly classified input instances by a classifier. Comput Biol Chem.

[CR34] Nath A, Subbiah K (2015). Maximizing lipocalin prediction through balanced and diversified training set and decision fusion. Comput Biol Chem.

[CR35] Nath A, Subbiah K (2015). Unsupervised learning assisted robust prediction of bioluminescent proteins. Comput Biol Med.

[CR36] Nath A, Chaube R, Karthikeyan S (2012) Discrimination of psychrophilic and mesophilic proteins using random forest algorithm. In: Biomedical engineering and biotechnology (iCBEB), 2012 international conference, 28–30 May 2012, pp 179–182. doi:10.1109/iCBEB.2012.151

[CR37] Nath A, Chaube R, Subbiah K (2013). An insight into the molecular basis for convergent evolution in fish antifreeze proteins. Comput Biol Med.

[CR38] Pérez-Cañamás M, Hernández C (2014). Key importance of small RNA binding for the activity of a glycine/tryptophan (GW) motif-containing viral suppressor of RNA silencing. J Biol Chem.

[CR39] Platt JC (1999) Fast training of support vector machines using sequential minimal optimization. In: Advances in kernel methods. MIT Press, pp 185–208

[CR40] Pugalenthi G, Kandaswamy KK, Suganthan PN, Archunan G, Sowdhamini R (2010). Identification of functionally diverse lipocalin proteins from sequence information using support vector machine. Amino Acids.

[CR41] Qu F, Morris TJ (2005). Suppressors of RNA silencing encoded by plant viruses and their role in viral infections. FEBS Lett.

[CR42] Senshu H (2011). A dual strategy for the suppression of host antiviral silencing: two distinct suppressors for viral replication and viral movement encoded by potato virus M. J Virol.

[CR43] Suvarna Vani K, Durga Bhavani S (2013) SMOTE based protein fold prediction classification. In: Meghanathan N, Nagamalai D, Chaki N (eds) Advances in computing and information technology, vol 177. Advances in intelligent systems and computing. Springer, Heidelberg, pp 541–550. doi:10.1007/978-3-642-31552-7_55

[CR44] Valli A, López-Moya JJ, García JA (2001) RNA silencing and its suppressors in the plant-virus interplay. In: eLS. Wiley doi:10.1002/9780470015902.a0021261

[CR45] Vapnik V (1995) The nature of statistical learning theory. Springer

[CR46] Vapnik V (1998). Statistical learning theory.

[CR47] Wang Y, Dang M, Hou H, Mei Y, Qian Y, Zhou X (2014). Identification of an RNA silencing suppressor encoded by a mastrevirus. J Gen Virol.

[CR48] Wei Q, Dunbrack RL (2013). the role of balanced training and testing data sets for binary classifiers in bioinformatics. PLoS ONE.

[CR49] Weiss GM, Provost F (2003). Learning when training data are costly: the effect of class distribution on tree induction. J Artif Int Res.

[CR50] Xiao J, Tang X, Li Y, Fang Z, Ma D, He Y, Li M (2011). Identification of microRNA precursors based on random forest with network-level representation method of stem-loop structure. BMC Bioinformatics.

[CR51] Xie H-L, Fu L, Nie X-D (2013). Using ensemble SVM to identify human GPCRs N-linked glycosylation sites based on the general form of Chou’s PseAAC. Protein Eng Des Sel.

[CR52] Youden WJ (1950). Index for rating diagnostic tests. Cancer.

