# Supplementary material for: Probing an optimal class distribution for enhancing prediction and feature characterization of plant virus-encoded RNA-silencing suppressors
Source: 3 Biotech. 2016 Mar 21;6(1):93. doi: 10.1007/s13205-016-0410-1 (PMC4801844; doi:10.1007/s13205-016-0410-1)
Supplement: Supplementary file 1 — Supplementary material 1 (DOCX 19 kb) [file 13205_2016_410_MOESM1_ESM.docx]

***SUPPLEMENTARY MATERIAL***

**Sample Sequence used to illustrate the extraction input feature vector**

>sp|A0AUJ5_P1|746-1070

SEELQPLHDDLSELKPLNVINNELIRQNMHITTLYSNMSKLQNDALATKAEMKLPLFGVAQLVVNQLKYNTTTHEWGERGDYVRKFVGKFFADFPTTQVPKQYMTRTTNGHIRITAYKALSLTSDPEIMMSRRMTQPMLTTAKQADCVFQSTTGATCTSASCTTNSSGVVLSNKCADPAPNTLRVRTMWDDIIIELPLQGGRVHVPLEGLCFSTIFLHMYLLVPDESVKLFHRTVTERAMPSLGQWPTLRHLATWVLNLVAMFPVLSTTPMPEILVHHESQSVHIPDCLGTATSGYHRLNIVTPYDFIIFATEIGRNGCQEYRVG

***Table S1:*** *Feature Vectors Component-1 for sample sequence*

| **Amino Acids** | **Amino Acid Composition** |
| --- | --- |
| **A** | *5.5385* |
| **R** | *4.9231* |
| **N** | *4.6154* |
| **D** | *4.0000* |
| **C** | *2.1538* |
| **Q** | *4.3077* |
| **E** | *4.9231* |
| **G** | *4.9231* |
| **H** | *3.6923* |
| **I** | *4.9231* |
| **L** | *10.7692* |
| **K** | *3.6923* |
| **M** | *4.0000* |
| **F** | *3.6923* |
| **P** | *5.8462* |
| **S** | *6.1538* |
| **T** | *10.7692* |
| **W** | *1.2308* |
| **Y** | *2.7692* |
| **V** | *7.0769* |

**Table S2:** Feature Vectors Component-2 & 3 for sample sequence

|  | ***Property Group Composition*** | ***Physicochemical-2grams*** |
| --- | --- | --- |
| Tiny amino acids group | *29.5385* | *38* |
| Small amino acids group | *51.0769* | *91* |
| Aliphatic amino acids group | *22.7692* | *17* |
| Non-polar amino acid groups | *52.9231* | *76* |
| Aromatic amino acid group | *11.3846* | *4* |
| Polar amino acid group | *47.0769* | *70* |
| Charged amino acid group | *21.2308* | *15* |
| Basic amino acid group | *12.3077* | *6* |
| Acidic amino acid group | *8.9231* | *4* |
| Hydrophobic acid group | *42.1538* | *53* |
| Hydrophilic acid group | *21.5385* | *14* |

**Table S3:** Feature Vectors Component-4 for sample sequence

| ***Dipeptide Counts*** | | | | | | | | | | | | | | | | | | | | |
| --- | --- | --- | --- | --- | --- | --- | --- | --- | --- | --- | --- | --- | --- | --- | --- | --- | --- | --- | --- | --- |
|  | **A** | **R** | **N** | **D** | **C** | **Q** | **E** | **G** | **H** | **I** | **L** | **K** | **M** | **F** | **P** | **S** | **T** | **W** | **Y** | **V** |
| **A** | *0* | *1* | *0* | *1* | *1* | *1* | *0* | *1* | *0* | *0* | *2* | *2* | *0* | *2* | *1* | *1* | *3* | *0* | *0* | *2* |
| **R** | *0* | *1* | *0* | *0* | *0* | *0* | *2* | *2* | *2* | *2* | *2* | *0* | *0* | *0* | *0* | *1* | *1* | *0* | *1* | *2* |
| **N** | *0* | *1* | *1* | *0* | *0* | *2* | *0* | *0* | *0* | *1* | *3* | *0* | *0* | *0* | *1* | *2* | *2* | *0* | *1* | *1* |
| **D** | *3* | *0* | *1* | *2* | *0* | *0* | *0* | *1* | *1* | *0* | *0* | *0* | *0* | *0* | *2* | *1* | *0* | *1* | *1* | *0* |
| **C** | *0* | *0* | *0* | *2* | *0* | *0* | *0* | *1* | *0* | *0* | *1* | *1* | *0* | *0* | *0* | *1* | *1* | *0* | *0* | *0* |
| **Q** | *1* | *1* | *1* | *0* | *1* | *0* | *0* | *1* | *0* | *0* | *3* | *2* | *0* | *1* | *0* | *1* | *2* | *0* | *0* | *0* |
| **E** | *1* | *0* | *1* | *1* | *0* | *1* | *1* | *1* | *2* | *1* | *1* | *0* | *0* | *0* | *2* | *2* | *2* | *0* | *0* | *0* |
| **G** | *0* | *1* | *2* | *0* | *0* | *1* | *1* | *1* | *0* | *1* | *2* | *0* | *0* | *1* | *0* | *2* | *1* | *1* | *0* | *2* |
| **H** | *0* | *1* | *0* | *0* | *0* | *0* | *0* | *1* | *1* | *0* | *2* | *0* | *1* | *1* | *0* | *0* | *1* | *0* | *1* | *3* |
| **I** | *0* | *1* | *1* | *1* | *0* | *0* | *3* | *0* | *3* | *3* | *1* | *0* | *0* | *1* | *0* | *0* | *1* | *0* | *0* | *1* |
| **L** | *2* | *1* | *1* | *1* | *1* | *2* | *4* | *1* | *1* | *1* | *1* | *3* | *1* | *1* | *5* | *2* | *3* | *0* | *1* | *3* |
| **K** | *1* | *1* | *1* | *0* | *0* | *0* | *0* | *1* | *0* | *0* | *2* | *0* | *1* | *0* | *1* | *1* | *1* | *0* | *1* | *1* |
| **M** | *2* | *1* | *2* | *0* | *0* | *0* | *1* | *0* | *1* | *1* | *0* | *0* | *1* | *0* | *2* | *0* | *1* | *0* | *1* | *0* |
| **F** | *0* | *0* | *0* | *2* | *1* | *0* | *0* | *0* | *0* | *2* | *2* | *2* | *1* | *1* | *0* | *0* | *0* | *0* | *0* | *1* |
| **P** | *1* | *0* | *0* | *2* | *0* | *2* | *0* | *0* | *0* | *1* | *2* | *1* | *2* | *2* | *0* | *0* | *2* | *1* | *0* | *3* |
| **S** | *1* | *0* | *1* | *0* | *0* | *2* | *2* | *0* | *0* | *0* | *4* | *0* | *2* | *1* | *1* | *1* | *3* | *0* | *1* | *0* |
| **T** | *5* | *3* | *2* | *0* | *2* | *0* | *0* | *1* | *0* | *2* | *2* | *0* | *2* | *0* | *2* | *3* | *9* | *0* | *0* | *2* |
| **W** | *0* | *0* | *0* | *0* | *0* | *1* | *1* | *0* | *0* | *0* | *0* | *0* | *1* | *0* | *0* | *0* | *1* | *0* | *0* | *0* |
| **Y** | *1* | *0* | *0* | *1* | *0* | *1* | *1* | *1* | *0* | *0* | *1* | *1* | *1* | *0* | *1* | *0* | *0* | *0* | *0* | *0* |
| **V** | *0* | *3* | *1* | *0* | *1* | *1* | *0* | *2* | *1* | *1* | *4* | *0* | *0* | *1* | *1* | *2* | *1* | *1* | *1* | *2* |

**TABLE S4a:** Statistical Paired t-test with the Percentage Correct Values

| Dataset | SMO  (Baseline Classifier) | NB | FLDA | IBK |
| --- | --- | --- | --- | --- |
| RSSP/NSP | 95.09 | 62.38* | 90.40* | 86.01* |
| *indicates significantly poor classifier than the baseline classifier | | | | |

**TABLE S4b:** Statistical Paired t-test with the AUC values

| Dataset | SMO  (Baseline Classifier) | NB | FLDA | IBK |
| --- | --- | --- | --- | --- |
| RSSP/NSP | 0.95 | 0.67* | 0.96 | 0.87 * |
| *indicates significantly poor classifier than the baseline classifier | | | | |

| Dataset | SMO  (Baseline Classifier) | NB | FLDA | IBK |
| --- | --- | --- | --- | --- |
| RSSP/NSP | 0.98 | 0.92* | 0.97 | 0.99 |
| *indicates significantly poor classifier than the baseline classifier | | | | |

**TABLE S4c:** Statistical Paired t-test with the TP rate

| Dataset | SMO  (Baseline Classifier) | NB | FLDA | IBK |
| --- | --- | --- | --- | --- |
| RSSP/NSP | 0.92 | 0.37* | 0.84* | 0.74* |
| *indicates significantly poor classifier than the baseline classifier | | | | |

**TABLE S4d:** Statistical Paired t-test with the TN rate
